# A new approach to a wicked problem: development of a cross-sector community-centered learning network to tackle childhood food inequity

**DOI:** 10.3389/fpubh.2024.1436760

**Published:** 2024-09-20

**Authors:** Aaron M. Schuh, Christopher Alexander, Kristen Gasperetti, Michelle C. Gorecki, Kimberly Cutler, Charles Hoffman, Robert S. Kahn, Chika Okano, Carley L. Riley

**Affiliations:** ^1^Division of Adolescent and Transition Medicine, Cincinnati Children’s Hospital Medical Center, Cincinnati, OH, United States; ^2^James M. Anderson Center for Health Systems Excellence, Cincinnati Children’s Hospital Medical Center, Cincinnati, OH, United States; ^3^Division of Critical Care Medicine, Cincinnati Children’s Hospital Medical Center, Cincinnati, OH, United States; ^4^Division of General and Community Pediatrics, Cincinnati Children’s Hospital Medical Center, Cincinnati, OH, United States; ^5^84.51°, Cincinnati, OH, United States; ^6^Department of Pediatrics, University of Cincinnati College of Medicine, Cincinnati, OH, United States; ^7^Michael Fisher Child Health Equity Center, Cincinnati Children's Hospital Medical Center, Cincinnati, OH, United States

**Keywords:** food insecurity, food equity, learning network, community-centered, improvement science

## Abstract

Food insecurity is a complex societal problem that disproportionately impacts households with children and those led by minoritized populations, with negative impacts on health across the life course. System to Achieve Food Equity adapted the learning systems model, used to address similarly complex problems, to tackle food insecurity at a neighborhood level. SAFE, born out of the COVID-19 pandemic, leverages a family-centered, community-based, cross-sector network fundamentally aimed at changing the food system so that all children in Cincinnati have the food they need to thrive. Through the following principles, Community-Led Network, Co-Production with Community, Equitable Sustainability, Learning to Learn Together, Distributing Leadership and Power, and Shared Data and Governance, SAFE has grown to over 300 individuals and 100 organizations, funded 9 novel interventions, distributed over 270,000 meals, and created a collaborative of motivated like-minded stakeholders. Future work includes improved data collection and sharing, support for increased stakeholder engagement and greater distribution of leadership and power, advocacy for policy change, refining measurement tools of network maturity for community settings, and collaboration with other efforts that contribute to food security indirectly.

## Introduction

Food insecurity, defined as a “lack of consistent access to enough food for every person in a household to live an active, healthy life,” (1) is a systemic, complex, and wicked (2) societal problem. Stakeholders across sectors have been working to find solutions, though not always collaboratively or with aligned goals. Like other wicked problems, food insecurity is symptomatic of larger systemic issues, including political, social, and logistical challenges, and will require robust action-oriented initiatives to make improvement.

Food insecurity is highly prevalent with 12.8% of households in the United States of America reporting being food insecure in 2022 (3). Food insecurity is not distributed evenly, with increased prevalence in households with children (17.3%), households with children headed by a single woman (33.1%) or a single man (21.2%), households with Black, non-Hispanic (22.4%) and Hispanic (20.8%) adults, and households with incomes below 185% of the federal poverty level (FPL; 32%) (3). Food insecurity in households with children tripled early in the COVID-19 pandemic (4), potentiating the ramifications of food insecurity that had already been exacerbated by societal inequities (5, 6). Its impact on children is broad, with negative effects on developmental, behavioral, academic, and emotional outcomes (7–10). It similarly affects health and well-being among adults, working nefariously across the life-course such that parents and caregivers living in food insecure households have worse mood, higher rates of depression, and increased rates of non-communicable disease (11–13).

Despite great efforts to improve food security on multiple levels (14–17), this wicked problem is likely to persist along with the associated negative outcomes if there are not efforts at systemic resolution (18). Herein, we describe the adaptation of a model to tackle similarly complex problems, the learning systems model (19, 20), made up of key elements including (1): a shared vision (2), collaborative methods whereby measures and actions are co-produced by system members (3), transparent data sharing that drives learning, research, innovation, planning, and priority setting (4), widespread capacity to change systems by applying continuous improvement methods (5), a reservoir of resources, information, knowledge, and know-how (6), a culture of trust, contribution, shared learning, curiosity, respect, and (7) governance structures, policies, and incentives aligned with inherent motivation to improve outcomes. This model has been utilized previously in community-based improvement endeavors (21–25), and informs the continued development of System to Achieve Food Equity (SAFE).

## Local context

Cincinnati is a city in southwestern Ohio, home to over 300,000 people, and the seat of Hamilton County. Cincinnati, like other large cities in the United States, has a history of racial segregation through redlining and additional racist policies that have concentrated disadvantage in distinct neighborhoods (26). While 27% of Cincinnati residents live below the FPL (27), some local neighborhoods have as many as 75% of people living below the FPL (28).

Before the COVID-19 pandemic, across Hamilton County, 90,250 people were food insecure, or 10.9% of the population, with an annual food budget deficit of over $58,000,000 (29). Children in Hamilton County under the age of 18 years were more likely to be food insecure (16.9%) compared to the general population (10.9%), as were Black (25%) and Hispanic (17%) residents compared to White (9%) residents (29). Focusing in on the city of Cincinnati, 31% of adults reported being food insecure (30), and among neighborhoods most impacted by food insecurity the rate (39%) was even higher (31). In 2017, 23% of parents and guardians in the Price Hill neighborhood of Cincinnatireported that their family experienced food insecurity in the past year (31).

There is a long history in Cincinnati of efforts to improve access and affordability of fresh and healthy food in the face of systemic challenges. Early programs such as Freestore Foodbank and The Greater Cincinnati Nutrition Council, established in 1971 and 1974 respectively, sought to improve access to food and promote self-reliance (32). More recently, in 2014, Produce Perks (33) came to Cincinnati, which works to increase access and consumption of healthy foods and supports local growers through doubling the value of Supplemental Nutrition Assistance Program (SNAP) benefits used to purchase fruits and vegetables. La Soupe (34) was also founded in 2014 and rescues otherwise wasted produce to create meals for food-insecure families. Additionally, the Greater Cincinnati Regional Food Policy Council was launched in 2015 with the mission to promote a healthy, equitable, and sustainable food system for all within Greater Cincinnati’s 10-county region (35). However, efforts to increase access have not always been successful. Since 1968, 82 farmer’s Markets have opened in the Cincinnati area, but only 30 remain (32). Additionally, these efforts to improve food security were often siloed away from each other, working without a city-wide shared vision and mission. Investments in Cincinnati’s food system continue, such as through the City of Cincinnati’s Office of Environment and Sustainability Urban Agriculture grants (36), but challenges to equitably transform the food system remain. A large gap persists in Cincinnati in access to affordable, healthy, and fresh food.

## Beginning of SAFE

Prior to the COVID-19 pandemic, food insecurity was a constant threat for many families and neighborhoods across Cincinnati, as well as the nation. The pandemic exacerbated these pre-existing inequities in food security (4), increasing the risk of adverse outcomes for children. In 2020, as school buildings across the city closed due to the pandemic, leaving many children without access to school-based nutrition programs, a diverse set of stakeholders from across sectors worked collectively to ensure children and families in Cincinnati had access to adequate nutrition. A team comprised of individuals from 84.51°, which is a retail data science company, and Cincinnati Children’s Hospital provided actionable data by mapping emergent and pre-existing food distribution sites across Cincinnati against neighborhood child poverty rates. These maps revealed inequitable distribution of emergency food resources with little to no access to food resources in multiple neighborhoods with anticipated high need. These data informed increased distribution of food resources into those areas of unmet need, with all stakeholders supporting the shared aim of ensuring food resources were available at least 3 days per week within one mile of every child in Cincinnati. From this broad-based emergency public health response emerged a group of highly engaged and invested stakeholders, including food banks, food pantries, data analysts, local grocers, a food policy council, and community members, motivated to build on previous food security efforts and collaboration that would lead to greater impact, innovation, and results. These stakeholders were initially brought together by a local city councilmember, but to continue efforts to reduce food insecurity beyond the pandemic, this group required further organization around a common purpose and collective action to tackle food system complexities. As such, Cincinnati Children’s Hospital Medical Center transitioned to the stewardship role of this coalition. The stakeholders wanted to emerge from the pandemic with a more effective and equitable food system utilizing the following principles adapted from the learning systems model: Distributing Leadership and Power, Community-Led Network, Co-Production with Community, Equitable Sustainability, Learning to Learn Together, and Shared Data and Governance. SAFE continues to refine and adapt the above principles into a family-centered, community-based, cross-sector learning network with a shared vision for all 70,000 children in Cincinnati to have the food that they need to grow, develop, learn, and thrive.

## Distributing leadership and power

**Figure 1:**
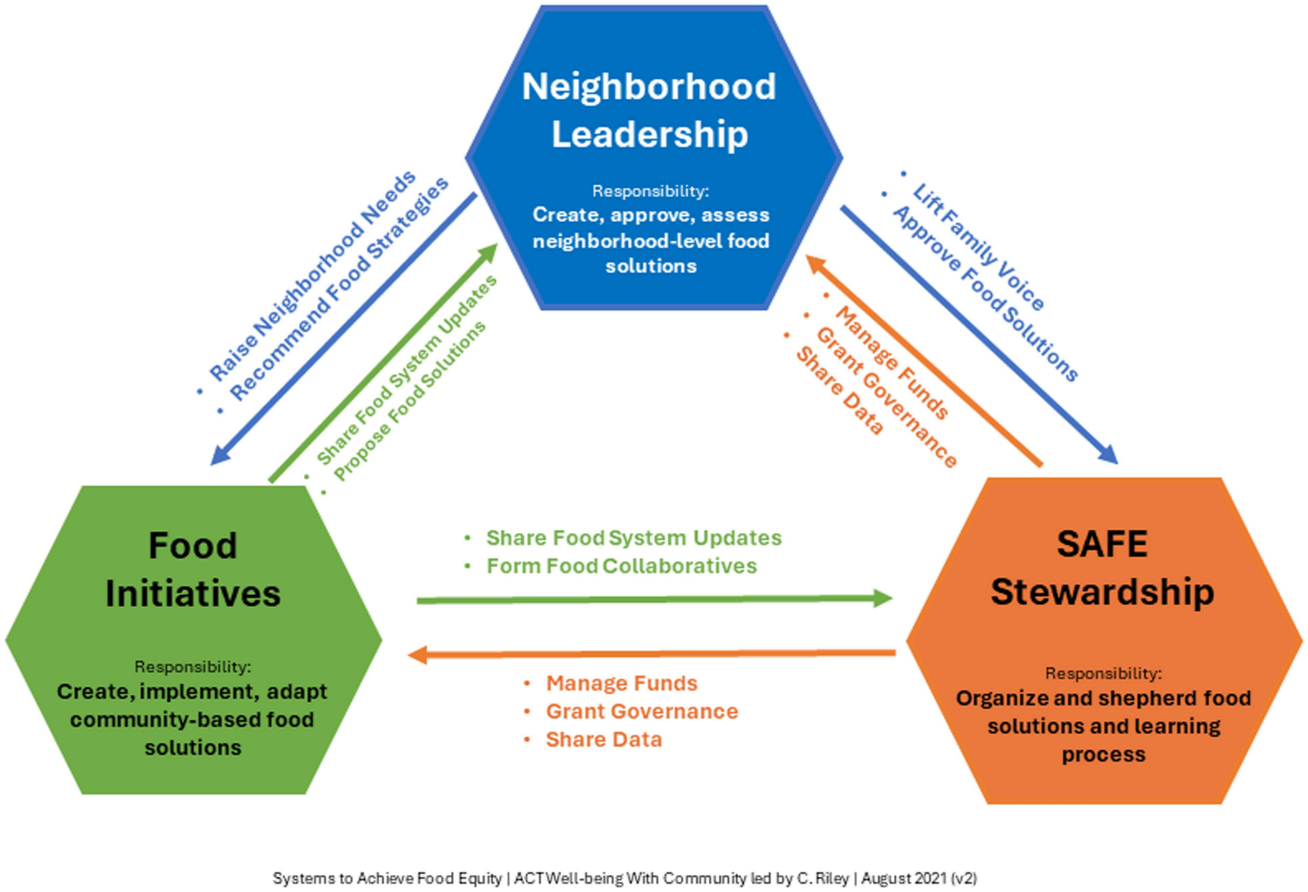
Model framework of interconnected nodes and distributed leadership.

SAFE has worked to distribute leadership across the network to reduce power differentials and encourage leaders to take ownership and responsibility of achieving our shared goal. Distributed leadership efforts include affirmation and skill-building of people who are not typically seen as leaders, democratization of collective decision-making around theory and goal setting, and co-design, selection, and evaluation of interventions. In three interconnected nodes (SAFE Stewardship, Neighborhood Leadership, and Food Initiatives), SAFE’s structure provides checks and balances with each node mutually reinforcing the others (Figure 1). Quarterly stakeholder surveys assess network growth, engagement, and opportunities for feedback, and identify stakeholders that want to increase or adjust their ownership within the network. As SAFE rapidly grows, stakeholder surveys have kept a pulse on the engagement of core members, led to leadership development conversations with interested members, and identified needs such as role clarification for leaders.

### SAFE stewardship

The Stewardship team provides operational oversight of the network and supports the development and execution of SAFE’s mission, vision, objectives, principles, and practices. The SAFE Stewardship team manages resource allocations and support based on what is proposed by community organizations in collaboration with the neighborhood leadership teams. It helps to manage funding, grants, and shared data. Roles within the Stewardship team include principal investigator or steward, community-based researchers, community engagement specialists, communication specialists, family partners, quality improvement leads, project managers, members of a local food policy council, and leaders from several food organizations. Through its early years, Cincinnati Children’s has served as a backbone organization, providing fundamental support of the network, including continuity of leadership, essential capacity for community engagement, data, project management, quality improvement, and research expertise, and funding for both members and initiatives.

### Neighborhood leadership

Each of the three engaged neighborhoods have developed or leveraged a Neighborhood Leadership team consisting of parents, caregivers, and community stakeholders. With these teams, SAFE aims to elevate the voices and leadership of people with lived experience and reallocate power to the neighborhood residents. Co-production of solutions with the neighborhood is vital to learning network success and the pursuit of equity (37). Neighborhood Leadership is also responsible for pinpointing neighborhood needs, recommending food strategies, and identifying, co-designing, and evaluating community-led initiatives to improve neighborhood food security.

### Food initiatives

Food Initiatives are responsible for creating, implementing, and adapting community-based food solutions. Additionally, they keep Neighborhood Leadership and SAFE Stewardship up to date on the food system and propose new food solutions while forming collaborations across the network. This group is comprised of community organizations that are part of Cincinnati’s food system, such as public schools, pantries, food hubs and distribution entities.

Distributing leadership and power aligns with learning system principles of collaborative methods, and governance structures and policies that align with inherent motivations to improve outcomes.

## Community-led network

SAFE is a network of diverse leaders with varied lived and professional expertise. Local community leaders are not only vital to informing the development and goals of SAFE, but also to selecting, supporting, and owning community-led interventions to improve food security in their own neighborhoods. Community-led interventions funded in the first year of SAFE included purchasing an indoor hydroponic farming system, supporting community gardens, establishing a mobile food pantry, delivery of boxes of shelf-stable food to families, opening of a community-designed, driven, and operated grocery store, providing delivery of prepared meals, community youth and family cooking education, and the creation of a farmer’s market (38). SAFE supports specific community-led interventions with robust financial support and quality improvement expertise, funded by the Kroger Company Zero Hunger | Zero Waste Foundation and Cincinnati Children’s Hospital Medical Center respectively, while building capacity of community members more broadly through quality improvement education, community engagement specialist involvement, and data and financial support to promote sustained future growth. Community-led network aligns learning system principles of a shared vision, widespread capacity to change systems, and aligning incentives with inherent motivation to improve outcomes.

## Co-production with community

Co-production with the community was fundamental to the creation and design of SAFE, as it is for SAFE’s continued impact and maturation. Co-production is used across learning health systems (39) and social innovations because the practice improves engagement and trust among all stakeholders, increases empowerment and satisfaction with services among local community members, and builds capacity and increases service utilization among organizations (40). Co-production is also an ethical practice, for communities deserve to participate in decisions that directly affect them (41). It was through intentional relationship development within the local community and among organizational stakeholders such as city government, food pantries and food banks, grocers, the public library and school system, and local non-profits that SAFE could effectively use co-production to lead from lived experience, co-design interventions, and reallocate power to those living in neighborhoods with high rates of food insecurity. Developing relationships required intentional effort to engage community members and stakeholders to understand their needs, wants, challenges and opportunities within the food system. Utilizing Cincinnati Children’s guidebook for equity in co-production (42), early SAFE leaders conducted 1-on-1 exploratory conversations and hosted a design session with diverse family and community partners. Co-production with the community continues to be lifted through multiple mechanisms, including monthly neighborhood leadership meetings and an annual SAFE network summit to reaffirm our shared aim, inform interventions, and ensure community presence and voice in all SAFE efforts. Co-production with community aligns learning system principles of collaboration and co-production with system members.

## Equitable sustainability

Food organizations within SAFE, which may have previously been attempting to access similar resources and thereby seen as competitors, had to create a shared mental model and collectively focus on improving outcomes for those they serve. As such, we had to shift from a scarcity and competition mindset to one that focused on effective, equitable distribution of shared resources. While individual organizations within SAFE manage their own resources, financial support for SAFE as a learning network has thus far come from Cincinnati Children’s Hospital Medical Center and The Kroger Company Zero Hunger | Zero Waste Foundation. We use data to distribute SAFE funding among neighborhoods to match proportional needs, and strategically partner with organizations to apply for external funding that supports both individual organizations and SAFE. Organizations within SAFE collaborated on successful applications for a planning grant and subsequent implementation grant from Feeding America. Additionally, individual organizations have leveraged data, pilot results, quality improvement, and relationships made possible through SAFE to apply for and receive funding from a wide range of funders, including national organizations. However, sustainable funding for SAFE is a persistent challenge. SAFE also supports food rescue and recovery as an ethical, environmentally sustainable, and effective model to reduce the meal gap. There is enough food in Cincinnati to feed everybody, but the systems in place provide inequitable access and distribution. As we move forward, we are increasing our focus on strategies and partnerships with organizations, such as a local food policy council, to create long-term sustainable solutions through policy and systems solutions. Whereas community-led interventions provide much needed meal gap coverage and learning, we can envision a system where affordable and fresh food is accessible to all through city planning, for example. Equitable sustainability aligns with learning system principles of creating a reservoir of resources and information, as well as development of a culture of trust and shared learning.

## Learning to learn together

SAFE utilizes improvement science (43) among all network stakeholders. It makes use of continuous quality improvement learning across the network through direct teaching at monthly stakeholder meetings, assigning dedicated quality improvement specialists to support community-led interventions, and hosting an annual SAFE Summit to share learnings. SAFE also partners with Cincinnati Children’s to participate in the biannual All Children Thrive (21) learning sessions and formal quality improvement training (44) for SAFE stakeholders.

**Figure 2:**
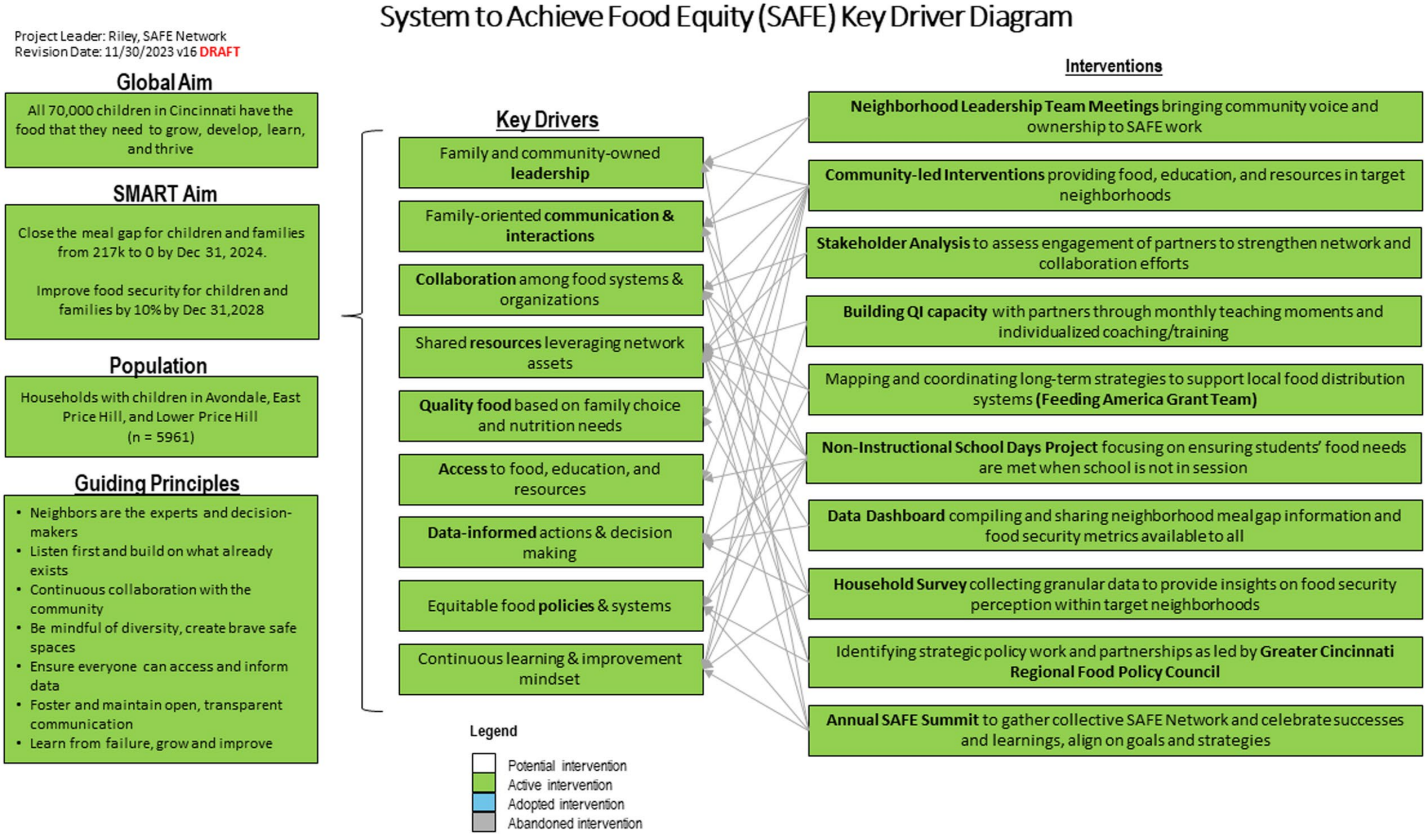
SAFE key driver diagram.

Our key driver diagram (Figure 2), a primary quality improvement tool that visualizes our change theory, was co-produced and iteratively refined with SAFE stakeholders. The SMART aims include closing the meal gap at a neighborhood level to zero by the end of 2024 and improving self-reported food security at a neighborhood level by 10% by the end of 2028. Key drivers include family and community-owned leadership, family-oriented communication and interactions, collaboration among food systems and organizations, shared resources leveraging network assets, quality food based on family choice and nutrition needs, access to food, education, and resources, data-informed actions and decision making, equitable food policies and systems, and continuous learning and improvement mindset. Learning to learn together aligns with learning system principle of widespread capacity to change systems by applying continuous improvement methods.

## Shared data and governance

Shared data are supported by data scientists and analysts from multiple industries and academic partners, including 84.51°, Cincinnati Children’s Hospital, Lehigh University, University of Cincinnati, and University of Louisville. Sharing data enables system-wide innovation, learning, planning, priority setting, quality improvement, and research. To inform network efforts robustly and accurately, SAFE needed to create a common language so all stakeholders could track progress together and build a public-facing democratized data dashboard so all stakeholders could readily access and utilize robust data. We collected data from public benefits programs, the public school system, and food organizations to build out a comprehensive representation of the local food system. Food organizations in SAFE measure their output in a variety of ways including pounds of food, number of calories in a box, and even food transaction costs. We created a common measure through a calculation based on multiple sources for the definition of a meal, such that a meal was equivalent to 1.2 pounds of food (45) or 600 calories (46) or 4.33 dollars (adjusted for inflation from 2021 estimates) (47). In this way, we were able to use all our available data to estimate neighborhood level meal gaps which is an estimation of the number of meals not covered by income, federal nutrition assistance programs, school meals, and charity food donations.

Our data infrastructure was co-designed and is publicly available as a data dashboard (48). These data have been used to assess progress over time, guide funding allocations across neighborhoods for SAFE community-led interventions, and provide neighborhood-specific information for a wide array of stakeholders. Shared data and governance aligns with the learning system principle of transparent data sharing that drives learning, research, innovation, planning and priority setting.

## Results

Since 2020, SAFE has grown in size and impact. SAFE has engaged over 300 individuals and 100 organizations across Ohio, Kentucky, and Pennsylvania. Our household survey from September 2022 to March 2023 across the three engaged neighborhoods revealed average food insecurity rates ranging from 85 to 91%. In their first year, nine funded interventions provided over 3,100 family touchpoints, 89,000 meals, 400 boxes of shelf stable food, 56,000 pounds of produce, 8,400 free grocery transactions, and 20 cooking classes (38). Through 2023, SAFE members were part of distributing over 270,000 meals, including over 190,000 in our engaged neighborhoods. The meal gap has fluctuated over time in our target neighborhoods, in part coinciding with the COVID-19 pandemic era support that has come and gone, ranging from 3 to 25% of meals unaccounted for across personal, governmental, and charitable resources in the most impacted neighborhood (48).

## Conclusion

There are many, complex drivers of food insecurity, with disproportionate impact on households with children and those identifying as racial/ethnic minorities (49), and with far-reaching effects on the health and well-being of those affected (7–9, 50). The learning systems model, as implemented by SAFE, brings together stakeholders to create change supported by shared data and improvement science. Early successes suggest that this model has promise in food equity.

Future work includes improved data collection and sharing within the network, support for increased stakeholder engagement and greater distribution of leadership and power, advocacy for policy change, refining measurement tools of network maturity for community settings, and collaboration with other resource accessibility efforts that contribute to food security indirectly. As SAFE continues to mature and increase impact, the network will rely on more stakeholders taking more ownership to promote change. Therefore, SAFE must ensure that stakeholders are engaged, have increasingly accessible and usable data, and be able to envision system change through policy and cross sector collaboration. SAFE’s successes and challenges since inception inform not only local improvement efforts, but also highlight what is outside of local control. What we cannot change ourselves is elevated to local policy makers and policy councils to advocate for system change. We anticipate that further maturation and scale of the network over the proceeding years, with a focus on improving the entire food system, will lead to broader, deeper, more transformational change that will fundamentally shift and improve the food system and its outcomes.

## Data Availability

The raw data supporting the conclusions of this article will be made available by the authors, without undue reservation.
